# The Effect of a Biostimulant Based on a Protein Hydrolysate of Rainbow Trout (*Oncorhynchus mykiss*) on the Growth and Yield of Wheat (*Triticum aestivum* L.)

**DOI:** 10.3390/molecules27196663

**Published:** 2022-10-07

**Authors:** Grigorii Alexandrovich Mironenko, Ivan Alexandrovich Zagorskii, Nataliya Anatolievna Bystrova, Konstantin Alexandrovich Kochetkov

**Affiliations:** 1 RM-Aquaculture LLC, Comintern srt., 7, Murmansk Region, Murmansk 183038, Russia; 2 A.N. Nesmeyanov Institute of Organoelement Compounds, Russian Academy of Sciences, Vavilova Str., 28, Moscow 119991, Russia

**Keywords:** biostimulant, rainbow trout protein hydrolysate, fish silage, spring wheat

## Abstract

The research object was the liquid protein hydrolysate “AGROMOREE” from the rainbow trout, which was provided by the company “Russian Aquaculture LLC”. The purpose of this study was the evaluation of the effect of the hydrolysate “AGROMOREE” as a biostimulant on the growth and yield of wheat (*Triticum aestivum* L.). Biometric indicators of wheat (*Triticum aestivum* L.) growth were determined in the laboratory and in field tests. In the laboratory, the liquid concentrated hydrolysate was dried to facilitate its use. “AGROMOREE” promoted an increase in germination of 2–4% in all samples compared to the control samples, as well as an increase in the length and number of wheat roots. The biostimulant “AGROMOREE” was introduced in the soil in liquid form at about 3000 L/ha and 4000 L/ha in the field tests. This study showed that “ARGOMOREE” contributed to an increase in the length and quantity of wheat ears, the quantity of grains in the ear and the seed weight. At the same time, the quantity of productive stems increased, so that the biostimulant “AGROMOREE” increased the productivity by 3.9–6.3% with respect to the control sample. In general, using the biostimulant “AGROMOREE” on spring wheat seeds from 2019 in the growing season of 2021 provided an increase in yield from 0.21 t/ha to 0.28 t/ha. The maximum value of raw gluten content in the seed was 5.2%, higher than the content in the control. The content of the mass fraction of protein in the seed was in the range of 12.33–12.56%, i.e., 2% higher than that of the control sample. Thus, according to qualitative and quantitative indicators and the total productivity indicators, the biostimulant «AGROMOREE» can be used to increase wheat productivity and reduce the use of nitrogen fertilizers.

## 1. Introduction

Over the last decade, the organic agriculture sector, focused on maintaining soil and plant fertility, has balanced food production and environmental conservation through the reuse of nutrients from organic waste nutrients. According to the Council of Europe Resolution 2019/1009 of 5 June 2019 (http://data.europa.eu/eli/reg/2019/1009/oj), organic waste is a natural biostimulant that can be classified according to its origin as follows: food waste, compost, manure, vermicompost, aquaculture waste (hydrolysates), seaweed extracts and wastewater treatment products [1,2]. All these natural biostimulants have a high potential to replace chemical fertilizers; therefore, research aimed at developing safe and environmentally friendly biostimulants, needed to reduce abiotic stresses and increase crop yields [3] should become a priority in the scientific community in the coming years.

Reducing the negative effects of environmental stresses by optimizing plant growth conditions through the supply of water, nutrients and growth regulators to plants [4,5] is important to increase crop productivity. Hydrolysates of vegetable proteins, rather than animal proteins, as growth stimulators are preferred to achieve this aim [6].

Currently, great attention is paid to biostimulants based on “sea food”: extracts of seaweed and protein hydrolysates obtained from the waste of commercial fishing or fish production in aquaculture. Recycling these wastes into valuable agricultural products used to improve plant growth and sustainability can further benefit the environment.

Seaweed has been used for many millennia to improve seed germination, plant growth, flower yields and fruit and berry production, increasing resistance to biotic and abiotic stresses. Recent studies have shown that the foliar application of seaweed extracts leads to increased growth and yield of crops such as wheat, winter rapeseed, strawberries, tomatoes, spinach, etc. Seaweed extracts have been proven to contain phytohormones such as auxins and cytokinins [7,8].

Aquaculture fish production worldwide reached more than 100 million tons in 2020, representing 50% of the total fish harvested, processed and marketed. The production of salmon fish, i.e., rainbow trout (*Oncorhynchus mykiss*) and Atlantic salmon (*Salmo salar*), is the most common in the European fish farming system. The fish processing plants produce a huge amount of by-products (about 35–70% of the total weight of salmon fish). In the process of growing and processing fish, a large amount of waste is collected, including spoiled fish, bone fragments (up to 37%), internal organs (up to 12%), skin, scales (up to 3%) and spoiled fillets (up to 45%). This material consists mainly of heads, trimmings, entrails and bones (fish silage) that need to be recycled to reduce the environmental pollution and improve the efficiency of agricultural production. The waste generation from aquaculture is a huge social problem and highlights a great necessity for an advanced practice to ensure the sustainable intensification of aquaculture processing. Fish silage has been already used as a fertilizer for ornamental plants and in horticulture and pasture fertilization. The liquefied fish products are used for breeding ornamental plants and planting trees in forests. Therefore, it would be relevant to use the silage to increase wheat production.

The most common method of disposal of salmon waste is the enzymatic or chemical hydrolysis of fish silage, that is, the production of fish protein hydrolysates (FPH) [9]. After preservation with formic acid (pH < 4), it is possible to use silage for fattening pigs and broilers [10] and to apply it in agriculture as a fertilizer. Hydrolyzed proteins contain numerous biologically active peptides with low molecular weight, having hormonal and immunological activity [11]. Since protein hydrolysates have chelating activity, they are able to reduce stress during plant growth, while improving the structure and quality of the soil. In addition, biostimulants increase the efficiency of conventional mineral fertilizers [12].

“Russian Aquaculture” LLC is the main company farming rainbow trout in aquaculture. In addition, the company is engaged in the processing and disposal of fish waste. Since 2017, it has been using rainbow trout protein hydrolysates (fish silage) as mink feed. The company is currently exploring the possibility of using fish silage as a biostimulant for crop production. Wheat (*Triticum aestivum* L.) is one of the most widely cultivated cereals in the world. However, the use of old seeds (second reproduction) is challenging. This research focused on the assessment of the possibility of using a biostimulant on old seeds to promote their germination and productive abilities.

The purpose of this study was to evaluate the use of the protein hydrolysate “AGROMOREE” from the rainbow trout as a biostimulant in laboratory and field research and to examine its impact on the growth and yield of wheat (*Triticum aestivum* L.). The general goal is to reduce or eliminate the use of chemical fertilizers by applying natural organic waste. In this respect, we also assessed the possibility of using the biostimulant on old seeds in order to promote their germination and productive possibilities.

## 2. Results and Discussion

### 2.1. Results of the Laboratory Tests

**Figure 1:**
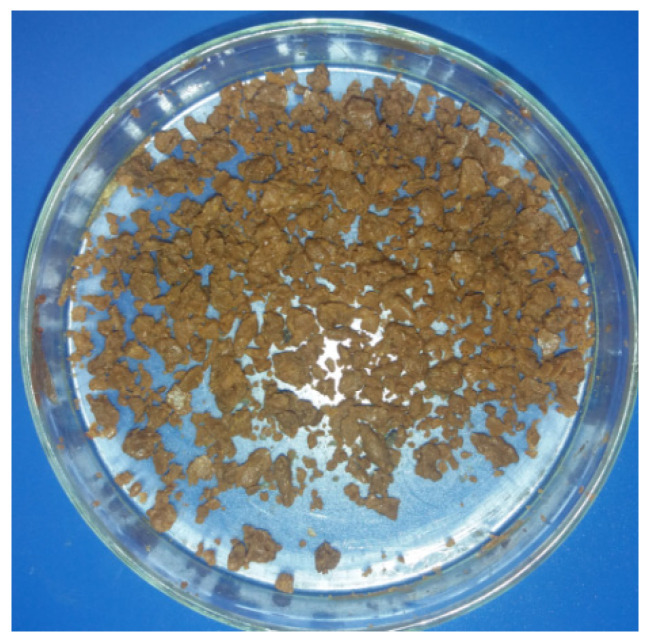
Dry hydrolyzate of rainbow trout “AGROMORE”.

The dry hydrolysate was used in the laboratory experiment (Figure 1). The content of total nitrogen (N) in the hydrolysate was 6.8%, which is consistent with the data of a Norwegian study [13], reporting a content of 6.25% of total N in dried samples. The dry hydrolysate “AGROMOREE” was placed into pots mixed with the soil in accordance with the N title [14]: 0.2 g (T_1_), 0.4 g (T_2_), 0.8 g (T_3_), 1.12 g (T_4_), 1.16 g (T_5_), 1.2 g (T_6_) of nitrogen per pot. The seeds, which were sown in the soil without the addition of the hydrolysate (T_0_) were used as the first reference sample. The second control sample consisted of seeds planted in a pot (T_7_) in which the soil was thoroughly mixed with a universal organic fertilizer containing 1.2 g of N. The results of the laboratory experiments on the use of the hydrolysate “AGROMOREE” as a biostimulant are shown in Table 1.

In this research, the wheat seeds (*Triticum aestivum* L.) “Trizo^®^” crop of 2018 were used. A slight increase in germination compared to the control (T_0_) was observed for all tested samples (T_1_–T_6_). The highest germination of 88% was observed in sample T_5_, which was 8% higher than the germination of the first control sample (T_0_) and 3% higher than the germination of the second control sample (T_7_). In samples T_2_–T_5_, an increase in the length and number of the roots were observed. Fish hydrolysates are known to improve (assimilation of nutrients) plant nutrient uptake and induce morphological changes in the root architecture [15]. The presence of a large number of strong roots and their increased length contribute to improving the vegetative growth. The short-chain peptides in the hydrolysate can have pseudo-hormonal and immune effects on plants [11]. Indeed, short-chain peptides can act as signaling agents to stimulate the endogenous production of auxin, which is used by plants to create neurotransmitters and hormones [6,11]. Ready-to-use amino acids from fish protein hydrolysates allow plants to save energy for protein synthesis. In addition, biostimulants reduce the period of plant adaptation after transplantation and during climatic stresses or diseases [16]. 

It is known that the Root length/Shoot height (R/S) ratio is one of the most important indicators for assessing plant health and can be used to evaluate the hormonal response to various types of stress caused by chemical, biological or physical agents [17]. The results of our calculations according to a previously described method [18] showed that the reaction of the plants to the “AGROMOREE” protein hydrolysate from the rainbow trout was not negative; therefore, this hydrolysate can be recommended as a biostimulant.

### 2.2. Results of the Field Research

According to the results of the laboratory observations, the plant developmental phases corresponded to those of the normal biological development of spring wheat plants in spring weather conditions. The determining factor in terms of productivity is the quantity of preserved plants that can be harvested, the density of the productive stems and productive bushiness (Table 2).

The height of the plants was in the range of 81.8–83.5 cm. In the experimental plots, 253 and 264 plants per m^2^ were obtained, in contrast to what observed for the control sample (240 pcs/m^2^). At the same time, the number of productive stems increased to 344 and 352 pcs/m^2^. Therefore, an increase in productivity (3.9–6.3% compared to the control sample) was observed when various doses of the biostimulant “AGROMOREE” were used.

The analysis of crop indicators showed that when the protein of hydrolysate ARGOMOREE was used, there was a slight increase the length of the ears, the quantity of spikelets (spicas) and grains in the ears, the weight of the grains from the ear (Table 3). The productivity of spring wheat in the control area was 2.22 t/ha. In general, the use of the biostimulator “AGROMOREE” on spring wheat in the growing season of 2021 provided an increase in harvest from 0.21 t/ha to 0.28 t/ha (Table 3).

The results of our research showed that the application the biostimulant “AGROMOREE” contributed to an increase in the productivity of spring wheat (the maximum increase was observed with the application of a dose of 4000 L/ha). The increase was 0.28 t/ha, i.e., 12.6% more in relation to the control area. The phytosanitary conditions of the crops with the application the “AGROMOREE” did not change, compared with those of the control crops. The appearance of the plants treated with the biostimulant “AGROMOREE” at the time of harvesting tended to improve.

The quantity of productive stems, the weight of the grains from the ears and the 1000-grain weight are the determining indicators of grain productivity. The mass of 1000 grains in the control sample was rather low, corresponding to 28.7 g, as expected for old grains. After using the “AGROMOREE”, it exceeded this value by 3.8–6.6%. Simultaneously with the increase in productivity, the application of the biostimulant “AGROMOREE” also contributed to improving the quality of the spring wheat grains. The crude gluten content varied from 25.2 to 26.5%. The maximum crude gluten content in the grains was found in conditions 3 (26.5%) and was higher (5.2%) than that in the control sample. Protein content is one of the most important indicators of wheat grain quality. The content of the mass fraction of protein in the grains in all conditions was in the range of 12.33–12.56% and increased by 2% compared to the control sample.

## 3. Materials and Methods

The object of the research was the liquid protein hydrolysate “AGROMOREE” obtained from the rainbow trout, which was provided by the company “Russian Aquaculture” LLC.

The muscles and tissues of rainbow trout consist of structural, myofibrillary and sarcoplasmic proteins that contain all the essential amino acids, in particular, lysine, phenylalanine and valine. They also contain endogenous enzymes such as pepsin, trypsin, chymotrypsin, collagenase and elastase. The protein hydrolysate, preserved with formic acid, consists of free amino acids and low-molecular-weight peptides. According to the analysis conducted with the Prominence HPLC system, a Shimadzu UV–Visible detector and the column ACEC18-AR, the most common amino acids contained in the “AGROMOREE” hydrolysate from the rainbow trout are serine (15.9%), glutamic acid (14.8%), lysine (9.4%), arginine (9.0%), glycine (7.2%) and leucine (6.2%) (the amino acid content is indicated as a percentage of the total amino acid content in the silage ≤ 2.0%). X-ray microanalysis indicated that the silage also contains up to 4.4 wt.% of sodium, 1.0 wt.% of magnesium, 0.2 wt.% of phosphorus, 1.0 wt.% of sulfur, 0.5 wt.% of potassium, 1.8 wt.% of calcium, 0.2 wt.% of iron. In general, the hydrolysate has a mass fraction of moisture ≤ 60.0%, a mass fraction of organic matter ≤ 15.0%, a mass fraction of ammonium nitrogen (NH4) 0.3%. Acidity indicates the activity of hydrogen ions (pH KCl); the hydrolysate pH was found to be not less than 3.5–4.0. These data are consistent with data previously reported [19].

### 3.1. Experiment № 1, Laboratory Tests

A preliminary laboratory test was conducted at the Nesmeyanov Institute of Organoelement Compounds of the Russian Academy of Sciences, Moscow, Russia, from May to October 2020. The geographical coordinates of the laboratory are 55°42′03 N. L.; 37°34′30 E. L.

The hydrolysate of fish protein “AGROMOREE” preserved with formic acid has a strong caviar odor and is not convenient for use as a fertilizer; therefore, it had to be dried before application, as described in the literature [13]. The drying process reduced its odor, facilitating its use as a biostimulant. A combined method of drying was used: in the first stage, 50 g of concentrated hydrolysate was subjected to thermal drying at a temperature of 100 °C for 4 h; in the second stage, drying was continued at room temperature for 48 h, leading to a loss of 70.5% of the original weight.

Spring wheat seeds (*Triticum aestivum* L.) of the Trizo^®^ variety of the second reproduction crop of 2018 were provided by the company “Zhito” LLC, Oktyabrsky district, Ryazan, Ryazan region, Russia, 54.609836° N. L., 39.80188° E. L. (https://zhitoecoproduct.ru). These seeds are registered in the State Register of Breeding Achievements of the Russian Federation No. 9908294. To exclude microbiological and fungal infection, the wheat seeds were sterilized in a 0.2% sodium hypochlorite solution for 10 min, washed three times with distilled water and dried in a desiccator at 30 °C for 48 h. The dried seeds were stored at 5 °C.

Laboratory experiments were performed on the seed (*Triticum aestivum* L.) in square pots (10 × 10 × 10 cm) in four replicates (growth chambers) with a phyto-LED UFO lighting-79-01-00 at a wavelength of Red 615/Blu 457 nm, providing illumination to the samples for 12/12 h at an intensity ≤ 250 lux, a temperature of 22.5 ± 1.50 °C and a relative humidity of 50 ± 1.5%.

The wheat seeds (*Triticum aestivum* L.) in the amount of 30 pieces were sown in eight plastic square pots containing universal soil provided by the company “Russian Lawns” LLC: 70% quartz sand, 23% silt and 7% clay. This soil has proven valid in testing various organic fertilizers. We placed 600 g of soil in each pot (550 g + 50 g to cover the seeds). Before the soil was put into the pots in accordance with a well-known method [14], it was thoroughly mixed with a portion of the dry hydrolysate “AGROMOREE”, containing 0.2 g (T_1_), 0.4 g (T_3_), 0.8 g (T_4_), 1.12 g (T_5_), 1.16 g (T_6_), 1.2 g (T_7_) of N (nitrogen). The seeds, which were sown in the soil without the addition of the hydrolysate (T_0_) were used as the first reference sample. The second control sample consisted of seeds planted in a pot (T_8_) in which the soil was thoroughly mixed with the organic universal fertilizer “Fishmeal” (from “M-Bi-Si” LLC) containing 1.2 g of N.

On the tenth day of the experiment, germination % (G% = [Number of germination seeds/Number of total seeds] ⋅ 100 (ISTA)) was determined. The length of the roots (R, cm) and the height of the shoots (S, cm) were determined after 14 days. For this, 15 shoots were carefully removed from each pot.

### 3.2. Experiment № 2, Field Experiment

The seeds of “Zlata” ^®^ of the second reproduction (The State Register of Breeding Achievements of the Russian Federation No. 9464217) were sown at the rate of 300 seeds/m^2^ in a sod-podzolic, heavy loamy soil (humus content–1.7%, nitrate nitrogen–7.0 mg/kg, ammonia nitrogen–1.8 mg/kg, pH of salt extract–5.3, mobile phosphorus–176 mg/kg, mobile potassium–198 mg/kg). Mineral fertilizers were added before sowing, i.e., azophoska 250 kg/ha (N40P40K40) (control) and the hydrolysate of fish protein “AGROMOREE”. The latter was manually applied in amounts of 3000 L/ha and 4000 L/ha, corresponding to 880 kg/ha and 1170 kg/ha of dry matter. The area of the experimental plots was 100 m^2^, the area of the research plots was 50 m^2^, and the number of replicates was four. The total area was divided into 12 equal strips, alternating and not in contact with each other. The scheme of the field diagram is a rectangle that was divided into squares, 4 horizontal and 3 vertical. Four graphs were prepared for each experimental setting and were then averaged.

The meteorological conditions of the spring period had a favorable effect on the germination of spring wheat. The precipitation and optimal temperature in May 2021 had a positive effect, promoting the production of harmonious shoots. The phases of tillering and tube releasing took place in relatively good conditions: the temperature regime in the II and III decades of June was characterized by moderately warm conditions with sufficient moisture, which contributed to the formation of additional stems in the spring wheat plants. The average monthly temperature in June 2021 was 22.3 °C, and 155 mm of precipitation fell. July 2021 was characterized by moderately warm temperatures with precipitation, which practically corresponded to the long-term average values. In the first decade of August 2021, the grain was in full ripening; at this time, there was warm weather with moderate humidification, and the air temperature was 21°C, with a precipitation of 20 mm.

#### 3.2.1. Crude Gluten Content in Grain

The content of gluten in the grain was determined according to a manual hand washing method [20,21]. The results are shown in Table 3.

#### 3.2.2. Mass Fraction of Protein

The protein content in the grain was determined by the Kjeldahl method as described [22,23]. The results are shown in Table 3.

### 3.3. Statistical Analysis

Statistical processing of the results was performed using Microsoft Excel software and STATISTICA 13.3 TRIAL (StatSoft Russia). The basic statistical parameters such as mean and standard deviation (SD) were computed, and one-way analysis of variance (ANOVA) was also carried out. The differences were considered significant at *p* ≤ 0.05.

## 4. Conclusions

The present study demonstrated the biostimulating effect of a protein hydrolysate of rainbow trout (*Oncorhynchus mykiss*) on the germination, growth and productivity of spring wheat (*Triticum aestivum* L.). It was found on the basis of qualitative and quantitative indicators, that the biostimulant “AGROMOREE” can be used to increase the yield and quality even of old grain. Using the biostimulant is of practical interest to stimulate plant growth and reduce the use of nitrogen fertilizers. Further research is needed to understand the mechanism of action of this biostimulant.

## Figures and Tables

**Table 1:** Biometric indicators of growth and development of wheat (*Triticum aestivum* L.) in the laboratory experiments.

	T_0_	T_1_	T_2_	T_3_	T_4_	T_5_	T_6_	T_7_
* G(%)	80.00 ^ns^	80.00 ″	82.00 ^ns^	83.80 ″	84.34 ″	88.00 ″	85.00 ^ns^	85.00 ″
* R(cm)	8.0 ″	8.7 ^ns^	9.48 **	11.0 **	10.9 ^ns^	11.69 ″	10.5 ″	10.7 ″
* S(cm)	10.2 ^ns^	11.1 ^ns^	12.0 ″	14.0 **	13.6 ″	14.8 **	13.3 ″	14.5 **
*** R/S(%)	100	99.92 ^ns^	100.72 ″	100.17 **	102.18 ″	100.65 **	100.7 ^ns^	94.08 ^ns^

* (G), Germination, (R), length of the roots, (S), height of the shoots; ** significant at *p* < 0.05; ″ statistically significant results at *p* < 0.01; ns, not significant; *** The change in biological endpoints in response to the treatment (exposure level) relative to the control group T_0_ was calculated as Response = µ_c_/μ_t_ × 100, where µ_c_ is the mean value of μ of the control group, and µ_t_ is the mean value of μ at each exposure level, such that the reference point is 100%.

**Table 2:** Biometric indicators of growth and development of wheat (*Triticum aestivum* L.) in the field tests.

№	Plant Height, cm	Plant Quantity, pcs/m^2^	Quantity of Productive Plant Stems, pcs/m^2^	Spica Length, cm	Quantity of Spica, pcs		The Mass of Grain from the Spica, g
					Spicas	Grains	
1. Control	81.8 ^ns^	240 ^ns^	331 ^ns^	5.6 **	23.0 ^ns^	23.8 **	0.85 ^ns^
2. “AGROMOREE”—3000 L/ga	82.5 **	253 **	344 **	5.8 **	23.5 ^ns^	24.3 **	0.88 ^ns^
3. “AGROMOREE”—4000 L/ga	83.5 *	264 *	352 *	5.9 **	24.3 ^ns^	24.5 **	0.90 **

* significant at *p* < 0.05; ** statistically significant results at *p* < 0.01; ns, not significant.

**Table 3:** Productivity and grain quality of spring wheat (*Triticum aestivum* L.).

	Productivity, t/ga	Increase of Productivity, t/ga	Mass of 1000 Grains, g	Gluten, %	Protein, %
1. Control	2.22 **	-	28.7 **	25.2 ^ns^	12.33 ^ns^
2. “AGROMOREE”—3000 L/ga	2.43 *	0.21	29.8 *	26.0 *	12.47 *
3. “AGROMOREE”—4000 L/ga	2.50 *	0.28	30.6 *	26.5 *	12.56 *

* significant at *p* < 0.05; ** statistically significant results at *p* < 0.01; ns, not significant.

## Data Availability

Not applicable.
